# Did Socioeconomic Inequality in Self-Reported Health in Chile Fall after the Equity-Based Healthcare Reform of 2005? A Concentration Index Decomposition Analysis

**DOI:** 10.1371/journal.pone.0138227

**Published:** 2015-09-29

**Authors:** Baltica Cabieses, Richard Cookson, Manuel Espinoza, Gillian Santorelli, Iris Delgado

**Affiliations:** 1 Faculty of Medicine Clínica Alemana, Universidad del Desarrollo, Chile, Av. La Plaza 680, Las Condes, Santiago, Chile; 2 Department of Health Sciences, University of York, Heslington, York, England, United Kingdom; 3 Centre for Health Economics, University of York, Heslington, York, YO10 5DD, England, United Kingdom; 4 Department of Public Health, Pontificia Universidad Católica de Chile, Marcoleta 340, Santiago, Chile; 5 Leeds Institute for Clinical Trials Research, University of Leeds, LS2 9JT, Leeds, United Kingdom; 6 Centro de Epidemiología y Políticas de Salud CEPS, Clínica Alemana—Faculty of Medicine,Universidad del Desarrollo, Chile, Av. La Plaza 680, Las Condes, Santiago, Chile; Leibniz Institute for Prevention Research and Epidemiology (BIPS), GERMANY

## Abstract

**Objective:**

Chile, a South American country recently defined as a high-income nation, carried out a major healthcare system reform from 2005 onwards that aimed at reducing socioeconomic inequality in health. This study aimed to estimate income-related inequality in self-reported health status (SRHS) in 2000 and 2013, before and after the reform, for the entire adult Chilean population.

**Methods:**

Using data on equivalized household income and adult SRHS from the 2000 and 2013 CASEN surveys (independent samples of 101 046 and 172 330 adult participants, respectively) we estimated Erreygers concentration indices (CIs) for above average SRHS for both years. We also decomposed the contribution of both “legitimate” standardizing variables (age and sex) and “illegitimate” variables (income, education, occupation, ethnicity, urban/rural, marital status, number of people living in the household, and healthcare entitlement).

**Results:**

There was a significant concentration of above average SRHS favoring richer people in Chile in both years, which was less pronounced in 2013 than 2000 (Erreygers corrected CI 0.165 [Standard Error, SE 0.007] in 2000 and 0.047 [SE 0.008] in 2013). To help interpret the magnitude of this decline, adults in the richest fifth of households were 33% more likely than those in the poorest fifth to report above-average health in 2000, falling to 11% in 2013. In 2013, the contribution of illegitimate factors to income-related inequality in SRHS remained higher than the contribution of legitimate factors.

**Conclusions:**

Income-related inequality in SRHS in Chile has fallen after the equity-based healthcare reform. Further research is needed to ascertain how far this fall in health inequality can be attributed to the 2005 healthcare reform as opposed to economic growth and other determinants of health that changed during the period.

## Introduction

Chile has achieved sustained economic growth since the 1980s and in May 2010 became a member of the Organization for Economic Co-operation and Development, which is traditionally considered to be a “rich country club” [1]. During President Ricardo Lagos´ term of office (2000–2006), Chile carried out a major healthcare system reform that aimed to reduce socioeconomic health inequality by improving the health status of the most deprived social groups [2]. This is the first study to examine whether socioeconomic health inequality in Chile changed before and after this equity-based healthcare reform.

### Background on the Chilean health care system

The Chilean healthcare system has experienced substantial changes over time. The publicly funded health care system, FONASA, was created in 1952 and dominated healthcare insurance until the military regime, 1973–1989. During the early 1980s, the military government undertook a series of measures to stimulate growth in membership of the private healthcare insurance system, ISAPRES. The policy rhetoric behind this reform focused on “individual freedom, justice (to give each one according to their own contribution), property rights, and subsidiarity” [3]. Since then, the Chilean healthcare system has been a mixed system characterized by segmentation. The private system covers about 25% of the wealthiest and healthiest population and the public sector covers around 60% of the population, including most of the disabled, sick and elderly. The public system is broadly divided into a 100% ‘free of charge’ service, available to those living below the means-tested poverty line, and the ‘public with co-payment’ service that varies in the proportion to be paid according to household earnings. The rest of the population is either part of the Army healthcare system (around 4%) or have no healthcare coverage at all (around 10%) [4,5]. With the exception of the public ‘free of charge’ provision that is given to the poorest in the country, everyone can choose between a range of healthcare insurance schemes, both public and private (the latter with over 2,500 different schemes available). On top of this, every person can choose to pay for private health insurance, which can come from a Chilean or international private insurer. Less information about this additional health coverage is known in Chile.

During President Ricardo Lago´s term of office (2000–2006), Chile carried out a new healthcare reform that aimed to reduce health inequities by improving the health status of the worst-off social groups. The rhetoric behind this reform focused on “the right to health, equity, solidarity, efficiency, and social participation” [2] and aimed at guaranteeing equal health and healthcare for all Chilean people according to their need and without discrimination (President Lagos national speech, 2000) [6]. The healthcare reform was implemented in 2003 and defined a set of health interventions that, according to the System of Health Guarantee´s Law, should be provided to every person that required them in Chile irrespective of type of provision entitlement, ability to pay, or any other non-need factor. The policy makers who designed and implemented this reform expected it to produce a significant impact on the population health [7]. This study aimed at examining whether socioeconomic health inequality in Chile changed before and after this equity-based healthcare reform.

### Introductory overview of the data

This study used comparable national survey data for two years– 2000 and 2013 –from the CASEN dataset from the Chilean Ministry of Planning. Our useable dataset comprised a large and nationally representative sample of Chilean adults: 101 046 in 2000 and 172 330 in 2013. We measured individual socioeconomic status using equivalized household income, and we measured individual health in terms of self-reported health status (SRHS).

SRHS has been reported as a significant risk factor for communicable disease [8], non-communicable illness [9] and mortality [10]. This has been found across countries [11,12], ethnic backgrounds [13] and age groups [14,15]. Hence, subjective SRHS is a widely used measure of health. Several individual factors are associated with SRHS, including demographic factors, socioeconomic status, material living conditions, and access to healthcare [16,17]. There is also a large empirical literature that has examined the relationships between population level SRHS and broader social, economic and political outcomes, such as economic development and income inequality [18–21].

Previous research on SRHS in other Latin American countries [22,23] supports the relevance of income and other dimensions of socioeconomic status as a key predictor of SRHS in Latin America. Subramanian et al. [24] used the Chilean CASEN survey 2000 and applied non-weighted multilevel analyses to explore the association between sub-national community income inequality and SRHS, allowing for individual level variables including income. The results indicated substantial income-related inequality in SRHS using interval groups of income with small groups at the very top and bottom of the social scale (what we have called an “extreme groups approach”). Vásquez et al. [25] measured income-related inequalities in health and health care utilization in the period 2000–2009 in Chile, including SRHS. They found that people in lower-income quintile groups report worse health status than people in higher income groups. The aim of this study was to update and refine this analysis of income-related health inequality using more recent data and more sophisticated methods for representing the gradient in income-related health inequality for the entire adult population living in Chile.

### Introductory overview of the concentration index methods

In this study our primary measure of inequality was based on the concentration index. The concentration curve plots the cumulative percentage of the health variable (y-axis) against the cumulative percentage of the sample, ranked by living standards, beginning with the poorest, and ending with the richest (x-axis) [26]. The basic concentration index is twice the area between the concentration curve and the line of equality (the 45-degree line) [27]. We then performed the Erreygers correction to allow for bias induced by the bounded nature of the SRHS outcome, which in our case of a binary variable simply means multiplying the CI by four times mean health [28–30]. We then performed adjustments to allow for factors such as age and sex which may be considered to represent “legitimate” sources of variation in health that are not unfair or unjust, to yield the “horizontal inequity index”. In order to understand what factors explained the observed pro-rich inequality in SRHS and the residual variation that was not explained by any of factors we conducted a decomposition analysis [31]. Wagstaff et al. [32] showed how the health concentration index can be decomposed into the contributions of individual factors to income-related health inequality, in which each contribution is the product of the sensitivity of health with respect to that factor and the degree of income-related inequality in that factor. There are different decomposition methods proposed in the literature (e.g. [32–34] and we use the Erreygers approach.

### Overview of the findings

We found that household income-related inequality in SRHS decreased between 2000 and 2013 but did not disappear. Results from this study contribute to knowledge about the current extent of income-related health inequity in Chile and comparable countries, including other Latin American countries and other countries with a similar level of economic development. The association with socioeconomic status and SRHS remains significant and strong, and factors such as education, income, occupation, and type of healthcare provision continue to be associated with an unequal income-related distribution of subjective health in Chile.

## Materials and Methods

### Purpose and research questions

This study aimed to answer the following research questions:

1. To what extent was above average SRHS in the adult Chilean population concentrated upon individuals with higher ranked equivalized household income in the years 2000 and 2013?
2. What were the absolute and relative contributions of legitimate and illegitimate determinants of income-related inequality in SRHS in 2000 and 2013?

### Population and sample

The CASEN survey (“Caracterización Socio-Económica Nacional”) provides information on the socioeconomic status of the population living in Chile. This before-after study is a secondary data analysis of the 2000 and 2013 CASEN surveys.

The CASEN surveys employ multistage probabilistic sampling, stratified by urban and rural area. Samples were independent from each other at the different time points, so this is a repeat cross section study rather than a longitudinal study. The sampling frame included every region in Chile. The inclusion criteria for selection of counties in both years were: (i) all urban counties with over 40 000 inhabitants, (ii) all rural counties irrespective of the number of inhabitants, (iii) a random selection of a small proportion of counties with less than 40 000 inhabitants. Fourteen and seventeen hard to reach counties were excluded from the 2000 and 2013 surveys respectively because of their very difficult geographical access. Within each county, households were randomly selected. Sampling included people living in transient camps in any of these counties, though these represented less than 1% of the total population of both surveys. No people living in institutions (e.g. hospitals, prison) were interviewed in any year [35].

In both years, data collection was via face-to-face interview by trained interviewers, using a validated questionnaire. The preferred respondent was the reported head of household, followed by their spouse or an adult household member. In most cases the head of the household and spouse provided the information about the household. Information was collected on all members of the household, including both adults and children [35].

The final sample in the full CASEN survey consisted of 252 748 people from a random sample of 65 036 households in 2000, and 218 491 people from a random sample of 64 842 households in 2013, representing around 95% of the total Chilean territory [36]. According to national statistics in both years, the mean number of households included in the CASEN was representative of the total population within each region and also representative of the population in each urban and rural setting from each region [37,38]. The response rate of the CASEN surveys was 80% in 2000 and above 85% in 2013 [35]. The most frequent reason for non-response, as reported by the Ministry of Social Development in the county that led the field work, is absence of any person in the household during working hours (nobody answered the door when the fieldworker called).

### Self-reported health status (SRHS)

Excluding missing values (n = 3344 out of the total sample of 252 748 participants, missing values representing 1.3%), a total of *101 046 participants* aged 15 and above in 2000 responded to the question: “From 1 to 5, how would you mark your general health status? Categories for responses in 2000 were: (1) very good, (2) good, (3) fair, (4) poor, (5) very poor.

Similarly, *172 330 participants* aged 15 and above in 2013 responded to the question: “From 1 to 7, how would you mark your general health status? (Excluding missing values, n = 2182 out of the total sample of 218 491 participants, missing values representing 0.9%). Categories for responses in 2013 were: (1) very poor, (2) poor, (3) regular, (4) fair, (5) good, (6) very good, (7) excellent.

Missingness on the SRHS question was below 1.5% in both samples; the larger sample size in 2013 was due to a reduction in the proportion of households with children under 15 in the full sample. The original SRHS variable in both years was a non-normally distributed bounded ordinal variable (range 1–5 in 2000 and 1–7 in 2013). We used the Erreygers approach to account for the bounded nature of the variable [29,30]. To avoid bias in comparisons over time due to the inflation of the scale between 2000 and 2013, we constructed a relative measure of health by dichotomizing SRHS as follows:

- For the CASEN 2000: “below average health” [categories 3–5] was represented by 0 (indicating below-average health i.e. worse than the mean SRHS response in year 2000 of 2.42) and “above average health” [categories 1–2] was represented by 1 (indicating above-average health).
- For the CASEN 2013: “below average health” [1–5] was represented by 0 (indicating below-average health i.e. worse than the mean SRHS response in year 2013 of 5.76) and “above average health” [6–7] was represented by 1 (indicating above-average health).

The use of a cut-off point around the mean response allows us to compare like with like between the two years [39]. Table 1 reports the proportions classified as “above average” health in each year, by age group. Reassuringly, the overall proportion was stable over time– 57% in 2000 and 58% in 2013 –and the age-specific proportions did not change much between the two years in any age group.

**Table 1:** Mean SRHS (range 0 “below average” to 1 “above average”) in years 2000 and 2013, by age groups and healthcare provision entitlement^*^.

	Year 2000			Year 2013			Change 2013–2000		
Age groups (years)	Fonasa (Public)	Isapres (Private)	*Overall*	Fonasa (Public)	Isapres (Private)	*Overall*	Fonasa (Public)	Isapres (Private)	*Overall*
15 to 24 “young adults”	0.77	0.82	*0*.*77*	0.77	0.85	*0*.*78*	0	0.03	***0*.*01***
25 to 44 “prime of life”	0.68	0.82	*0*.*68*	0.64	0.76	*0*.*67*	-0.04	-0.06	***-0*.*01***
45 to 64 “middle age”	0.43	0.68	*0*.*44*	0.44	0.61	*0*.*47*	0.01	-0.07	***0***
65 to 74 “late middle age”	0.33	0.58	*0*.*32*	0.34	0.50	*0*.*36*	0.01	-0.08	***0*.*04***
75+ “elderly”	0.29	0.47	*0*.*25*	0.29	0.44	*0*.*30*	0	-0.03	***0*.*05***
**All ages**	**0.57**	**0.78**	***0*.*57***	**0.61**	**0.75**	***0*.*58***	**0.04**	**-0.03**	***0*.*01***

*Other provision and none/don´t know excluded from this table as they represent small numbers and are more difficult to interpret in terms of social gradients

### Equivalized household income

We used the OECD-modified scale of equivalized household income for both years (continuous variable). This is the standard approach to measuring income in the literature, both in the literature on socioeconomic health inequality and in the more general literature on income inequality. The reason for using household income is that income is shared within households and so individual income yields a misleading impression of the standard of living e.g. of people who are looking after children but have high earning spouses. The reason for equivalization is to allow for household economies of scale–for example, cooking a meal for four people costs less than cooking four separate meals for one person. This scale was first proposed by Haagenars et al. [40] and adopted by the Statistical Office of the European Union (EUROSTAT) in the late 1990s. It assigns a value of 1 to the household head, 0.5 to each additional adult member and 0.3 to each child.

### Legitimate and illegitimate factors

**Fig 1:**
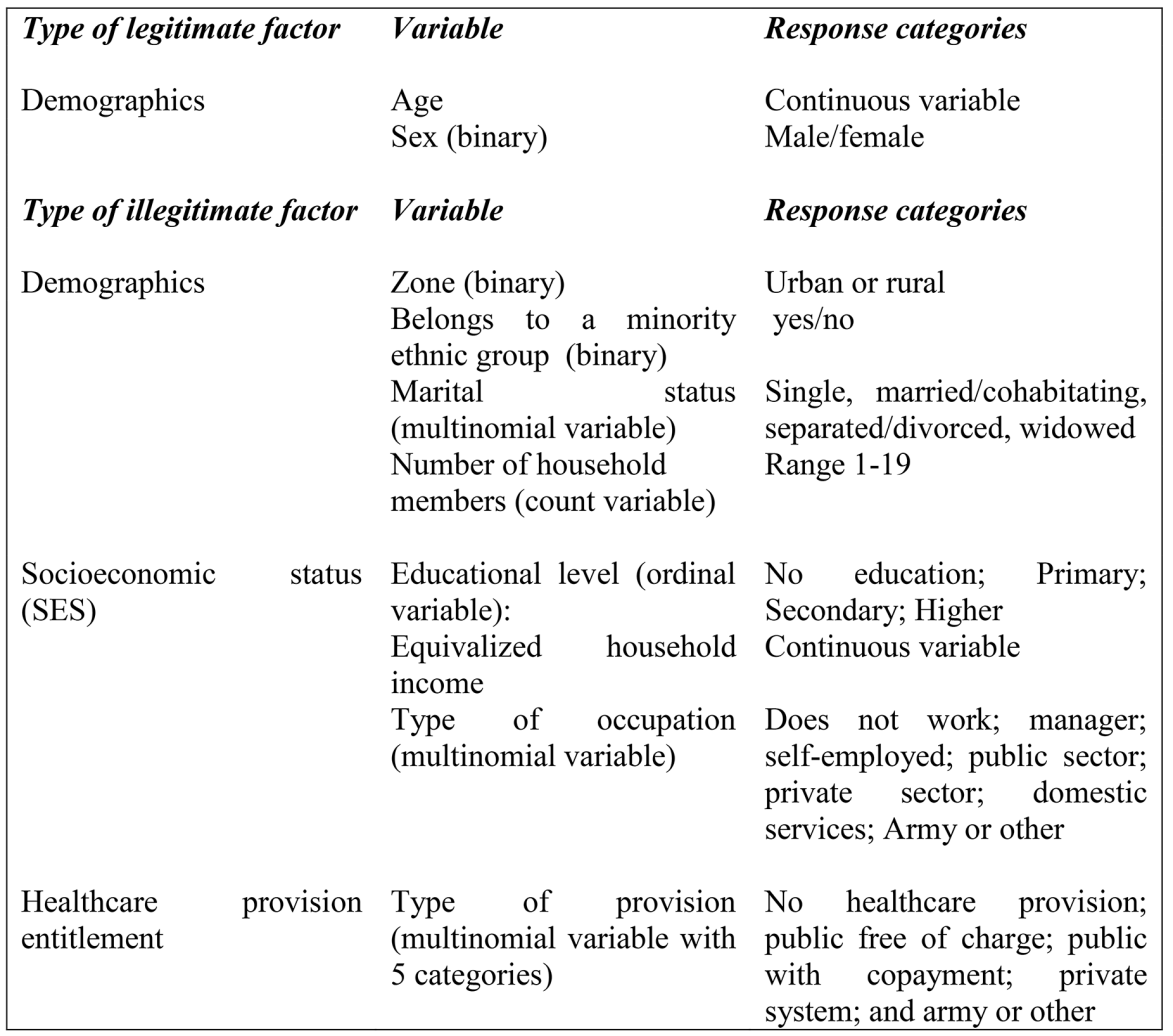
Summary of study variables included as legitimate and illegitimate factors for inequality in SRHS, CASEN surveys 2000 and 2013.

There are many possible determinants of income-related variation in health, which can be categorized into “legitimate” and “illegitimate” determinants [27,41]. This is always a normative categorization requiring potentially contestable value judgment. Legitimate determinants are sources of variation in health that are not considered to be “unfair” or “unjust”, such as age and, more controversially, sex. Illegitimate determinants by contrast are sources of variation in health that are considered to be “unfair” or “unjust”, such as dimensions of socioeconomic status (SES) or other closely related characteristics like ethnicity. All of these diverse dimensions of SES [42] are consistently and strongly associated with health status [43]. Fig 1 presents all legitimate and illegitimate factors included in this study. Missingness in these factors in both CASEN surveys were below 0.5%. Only cases with complete covariate data were included in the final analysis. We analysed two scenarios: age and sex as the only legitimate factors, and a longer list of illegitimate factors including household size and marital status. We found no significant differences between these two analytical approaches and therefore we present the full list of factors in this manuscript.

### Analysis

#### Weighting strategy

Data analyses were conducted with the STATA 13 statistical software package [44] and estimations were weighted to take into account the complex multistage sampling strategy of both surveys [45].

#### Inequality ratio and inequality gap

Since concentration indices can be hard for policy makers to interpret, we also perform a simpler analysis based on more intuitive ratio and gap measures of inequality. We included estimates of the inequality ratio and inequality gap, based on equivalized household income quintiles. The income inequality ratio (20:20) is the relative difference in mean health between top and bottom income quintile groups (twentieths), whereas the income inequality gap (20–20) is the absolute difference in mean health between these two extreme groups. We estimated these for years 2000 and 2013 separately, and compared the change in 2013–2000.

#### Concentration curves

The concentration curve provides a means of assessing the degree of income-related inequality in the distribution of a health variable. The concentration curve plots the cumulative percentage of the health variable (y-axis) against the cumulative percentage of the sample, ranked by living standards, beginning with the poorest, and ending with the richest (x-axis) [26]. We estimated the concentration curves for income related SRHS in years 2000 and 2013.

#### Concentration Index (CI) and Erreygers correction

The concentration index (CI) is defined as twice the area between the concentration curve and the line of equality (the 45-degree line) [46,47]. If the health variable represents a positive health state like “above average” SRHS, a positive value of the CI means that good health is higher among the rich. The CI is a rank-weighted sum of the health of the population, bounded between –1 and 1. The value of the CI is not a ratio scale measure of the magnitude of inequality but only enables inequality to be compared in an ordinal sense–for example, a value of 0.4 indicates more inequality than a value of 0.2, but does not represent “twice as much” inequality.

We estimated two different versions of the CI (and its standard error, SE). First, we estimated the uncorrected CI (SE) using the convenient regression method corrected for within cluster correlation and potential serial correlation [47].

Then, we computed the corrected Erreygers CI [28] which standardizes the uncorrected index by adjusting the CI in order to allow for the bounded nature of the health variable. In this context of a binary health variable, the Erreygers CI is the CI multiplied by four times the mean health or outcome of interest [28]. This estimate produces an index that satisfies various attractive axiomatic properties for an inequality index, including the sign condition, scale invariance and mirror properties [29].

Standardized estimation of the uncorrected CIs: Horizontal inequity indexes

There is substantial existing literature reporting uncorrected concentration indices, prior to the development of the corrected versions. To facilitate comparison with this previous literature, we report uncorrected results as well as corrected results [30]. We standardize both corrected and uncorrected indices to adjust for differences in legitimate factors [48] such as age and sex in order to produce a “horizontal inequity index” that indicates health inequality attributable to illegitimate factors. This index is computed as twice the area between two different concentration curves: one shows the actual cumulative health share of each income group and another one shows the “expected” cumulative health share given legitimate factors [49].

The “indirect” standardization method (or “*fairness gap”*) was estimated through the deviation of the actual distribution from a “fair” one driven by legitimate determinants alone, in which all illegitimate differences have been removed [49]. The “direct” standardization method (or “*direct unfairness”*) adds the variation generated by illegitimate determinants to mean health, that is, it eliminates legitimate differences in health [32,42].

#### Decomposition of the Erreygers CI

We continued the analysis using the Erreygers´ decomposition method [28]. In order to understand what factors explained the observed pro-rich inequality in SRHS, and also the residual variation that was not explained by any of factors, we conducted a decomposition of the Erreygers CI into legitimate and illegitimate factors [31]. We repeated the same analysis using the unstandardized CI to assess changes due to correction methods of the CI and there was almost no difference between them. We added age-squared in the decomposition to improve the fit of the data. Similar to other decomposition methods, the Erreygers CI decomposition is based on general regression analyses [28], as follows:

- (a)
  $$C\;=\;\hat{\beta }\frac{\bar{\gamma }}{\bar{h}}{\hat{C}}_{y}+\sum_{j}{\hat{\gamma }}_{j}\frac{{\bar{Z}}_{j}}{h}\hat{C}z_{j}+\sum_{k}{\hat{\delta }}_{k}\frac{{\bar{x}}_{k}}{h}\hat{C}x_{k}+\frac{{GC}_{\hat{\varepsilon }}}{\bar{h}}$$
  Where $\hat{\beta },\;{\hat{\gamma }}_{j},\;{\hat{\delta }}_{k}$ are OLS estimates, ${\hat{C}}_{w}$ are estimated concentration indices, $\bar{w}$ are sample means and ${GC}_{\hat{\varepsilon }}$ is the generalized concentration index of residuals [49].

This decomposition analysis provides more detailed information than standardized CI, since it estimates the specific contributions to income-related health inequality of each legitimate and illegitimate factor [30]. Results of decomposition analysis are often presented in tornado graphs. Given the cross-sectional nature of the datasets, this analysis does not allow causal inference, but for the identification of associations and particular contributions to inequality in SRHS in 2000 and 2013.

#### Ethical guidelines

This work was conducted using an anonymous nationally representative dataset available for free at the Ministry of Social Development secured Web Page after completion of an online form (http://observatorio.ministeriodesarrollosocial.gob.cl/casen/casen_obj.php). Since data was completely anonymous, informed consent was not necessary. We conducted secondary data analysis and therefore no particular individual can be identified through this work. Our study follows the Declaration of Helsinki and Singapore standards.

## Results and Discussion

### Results

#### Descriptive statistics

Descriptive statistics appear in Table 2. The mean age of the Chilean population was 30.6 years [95% CI 30.47–30.80] in 2000 and 35.3 years [95% CI = 35.1–35.5] in 2013 (p<0.001). Just over half were women in both years (51.1% in 2000 and 52.6% in 2013) and only about 12% of the sample lived in a rural zone in both time points (p>0.05). Half of the sample reported single marital status in both 2000 and 2013 (p>0.05), followed by married status (around 40% in both years) (p>0.05). The mean number of household members was 4.7 [95% CI = 4.6–4.7] in year 2000 and 4.1 [95% CI = 4.0–4.1] in 2013 (p<0.001). A significantly larger proportion of the sample reported belonging to a minority ethnic group over time (4% in 2000 versus 9% in 2013) (p<0.001). When comparing both years, people with “above average” SRHS were not significantly older over time (mean 37 years old in both years, p>0.05). Over time people reporting “above average” SRHS were also less likely to be women (60.6% in 2000 versus 49.7% in 2013) (p<0.001), more likely to live in rural areas (11% in 2000 versus 12.5 in 2013) (p<0.05), and more likely to be single (29.4% in year 2000 versus 44.8% in year 2013) (p<0.001).

**Table 2:** Descriptive statistics of variables in this study, crude and stratified by “above average” (i.e. above-average) SRHS.

	YEAR 2000		YEAR 2013	
	Total sample %/Mean (95%CI)	“Above average” SRHS [Adult sample only] %/Mean (95%CI)	Total sample %/Mean (95%CI)	“Above average” SRHS [Adult sample only] %/Mean (95%CI)
**Demographics:**				
Age (continuous)	X = 30.63 (30.47–30.80)	X = 37.34 (37.51–37.98)	X = 35.38 (35.16–35.59)	X = 37.74 (37.50–37.98)
Sex (female)**	51.08 (50.67–51.50)	60.63 (59.92–61.54)	52.63 (52.33–52.93)	49.69 (49.20–50.18)
Zone (rural)*	12.90 (12.76–13.05)	10.98 (10.73–11.23)	13.15 (12.80–13.51)	12.50 (12.09–12.91)
Marital status:				
Single**	51.23(50.81–51.64)	29.41 (28.68–30.14)	50.42 (50.05–50.80)	44.81 (44.22–45.43)
Married/ cohabiting**	41.21 (40.80–41.62)	60.76 (59.95–61.55)	39.70 (39.28–40.11)	46.16 (45.49–46.84)
Divorced/separated**	3.80 (3.65–3.95)	5.84 (5.42–6.30)	5.62 (5.61–5.83)	5.95 (5.61–6.31)
Widow*	3.76 (3.63–3.90)	3.99 (3.69–4.32)	4.26 (4.11–4.41)	3.07 (2.89–3.26)
Number of household members (continuous)*	X = 4.69 (4.68–4.71)	X = 4.27 (4.24–4.29)	X = 4.10 (4.07–4.14)	X = 4.06 (4.02–4.10)
Belong to an ethnic group in Chile (yes)	4.51 (4.37–4.65)	3.87 (3.63–4.13)	9.14 (8.78–9.53)	8.41 (7.92–8.92)
**Socioeconomics:**				
Mean equivalized household income ($USD for each year***):				
Poorest quintile 1**	X = 111.95 (111.38–112.51)	X = 113.16 (111.77–114.56)	X = 189.71 (188.37–191.05)	X = 192.61 (190.52–194.69)
Quintile 2**	X = 224.86 (224.37–225.36)	X = 226.11 (225.16–227.04)	X = 362.10 (361.19–363.00)	X = 363.60 (362.40–364.80)
Quintile 3**	X = 351.60 (350.71–352.49)	X = 352.23 (350.74–353.72)	X = 544.86 (543.68–546.08)	X = 546.70 (544.97–548.43)
Quintile 4**	X = 566.41 (564.38–568.44)	X = 571.90 (567.70–576.09)	X = 834.06 (831.86–836.26)	X = 834.19 (831.13–837.82)
Wealthiest quintile 5**	X = 1 820.26 (1 748.08–1 892.44)	X = 1 831.68 (1 715.34–1 948.03)	X = 2 100.43 (2 203.08–2 228.31)	X = 2 279.99 (2 191.04–2 368.83)
Type of occupation:				
Does not work**	63.69 (63.29–64.10)	51.22 (50.40–52.05)	58.35 (57.99–58.71)	43.72 (43.16–44.28)
Manager**	1.44 (1.32–1.58)	2.16 (1.83–2.56)	0.81 (0.74–0.89)	1.09 (0.68–1.21)
Self employed	7.18 (6.98–7.13)	9.99 (9.45–10.55)	8.11 (7.85–8.38)	9.42 (9.02–9.84)
Public sector	3.89 (3.69–4.09)	6.30 (5.77–6.87)	4.22 (4.05–4.39)	5.78 (5.52–6.05)
Private sector**	20.64 (20.30–20.97)	25.76 (25.04–26.48)	26.65 (26.30–27.01)	37.84 (37.26–38.43)
Domestic services**	2.11 (2.00–2.22)	3.09 (2.83–3.38)	1.57 (1.49–1.66)	1.67 (1.55–1.81)
Army or other*	0.50 (0.45–0.56)	0.66 (0.54–0.80)	0.29 (0.25–0.39)	0.47 (0.40–0.55)
Educational level:				
No education**	10.13 (9.92–10.35)	2.49 (2.30–2.69)	11.37 (11.12–11.62)	1.57 (1.42–1.73)
Up to primary level**	40.70 (40.30–41.09)	24.32 (23.75–24.90)	30.49 (30.09–30.89)	17.78 (17.26–18.32)
Up to secondary level*	26.66 (26.29–27.03)	37.77 (36.97–38.59)	31.55 (31.12–31.99)	40.38 (39.71–41.06)
Higher **	22.51 (22.12–22.91)	35.42 (34.59–36.27)	26.59 (26.14–27.04)	40.27 (39.88–40.99)
**Healthcare provision entitlement:**				
No health care provision**	0.92 (0.82–1.04)	0.66 (0.55–0.79)	1.82 (1.70–1.95)	1.90 (1.74–2.07)
Public free (means tested)**	24.28 (23.99–24.57)	19.42 (18.93–19.93)	28.04 (27.43–28.66)	22.90 (22.26–23.56)
Public with co-payment**	41.56 (41.15–41.96)	41.36 (40.56–42.16)	50.69 (50.02–51.36)	51.42 (50.60–52.23)
Private**	30.04 (29.62–30.47)	34.94 (34.09–38.50)	16.52 (15.96–17.08)	20.56 (19.82–21.32)
Army or other	3.20 (3.06–3.35)	3.62 (3.33–3.94)	2.93 (2.74–3.13)	3.22 (2.97–3.49)

*Chi2 test or t-test p value <0.05 at 95% confidence level when comparing categories of “above average” SRHS between year 2000 and 2013

**Chi2 test or t-test p value <0.001 at 95% confidence level when comparing categories of “above average” SRHS between year 2000 and 2013

***Currency exchange rates obtained from the International Monetary Fund web source (available in: http://www.imf.org/external/np/fin/data/param_rms_mth.aspx)

In both years, the most reported type of occupation among those with a formal job in the total population was in the private sector (20.6% [95%CI = 20.3–20.9] in year 2000; 26.6% [95%CI = 26.3–27.0] in 2013), followed by self-employed and working in the public sector (around 7% and 4% in both years, respectively). In year 2000, 10.1% reported no education at all, 40.7% reported up to primary, 26.6% secondary level and 22.5% higher education level. In year 2013, 11.3% of the sample reported no education, 30.4% reported up to primary level only, 31.5% secondary level, and 26.5% higher education level. In year 2000, 24.3% [95%CI = 23.9–24.5] reported public free of charge provision, 41.5% [95%CI = 41.1–41.9] public with copayment, 30.0% [95%CI = 29.6–30.4] private and 0.9% [95%CI = 0.8–1.0] no healthcare provision at all. The rest of the sample (3.2%) reported army or other type of healthcare provision entitlement. In year 2013, just over half of the total population reported public with co-payment healthcare provision entitlement (50.1% [95%CI = 50.0–51.3]), followed by public free of charge (28.0% [95%CI = 27.4–28.6] and the private system (16.5% [95%CI = 15.9–17.1]. Less than a 3% reported army or other type of provision (2.9% [95%CI = 2.7–3.1] and 1.8% reported no healthcare provision entitlement at all [95%CI = 1.7–1.9].

#### Self-reported health status (SRHS)

**Fig 2:**
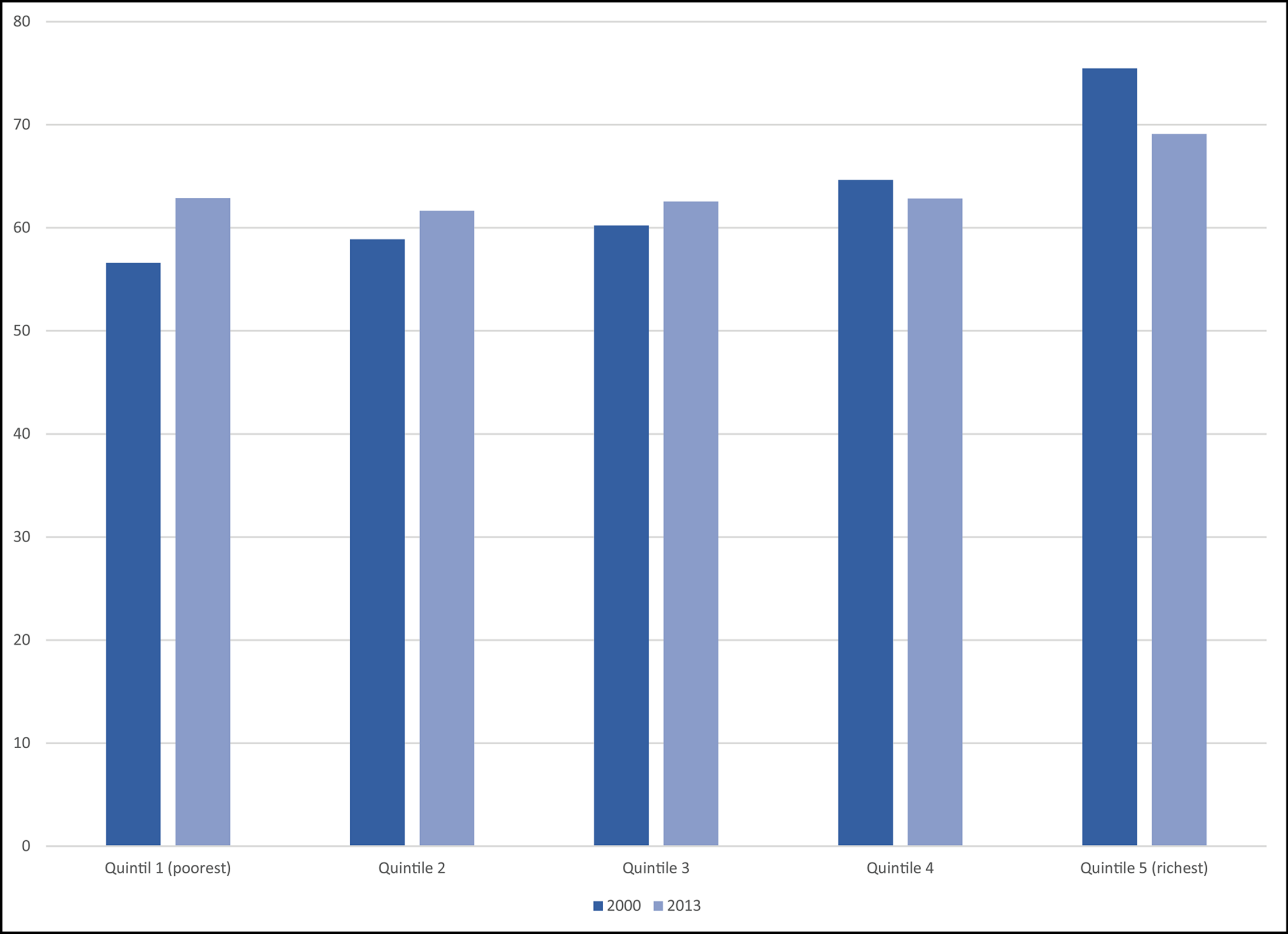
Proportion of the adult population reporting “above average” SRHS within each equivalized household income quintile in year 2000 and 2013.

Fig 2 displays the proportion of the population in Chile with “above average” health by socioeconomic status in 2000 and 2013. This figure shows the relative reduction in the gap between extreme equivalized household income groups between 2000 and 2013, as there was an increase in the proportion of people with “above average” SRHS in the poorest income quintile in 2013 compared to 2000; whereas there was a reduction in the proportion of people reporting “above average” SRHS in the top income quintile group in 2013 compared to 2000.

We also explored the mean value of SRHS (range 0 “below average” to 1 “above average”) in years 2000 and 2013, by age groups and healthcare provision entitlement (Table 1) and compared the absolute change in each category between 2013–2000. We observed differences in SRHS by provision and age groups in both years separately, but found a general increase in mean SRHS in almost all age groups and healthcare provision groups, with the exception of the 25 to 44 years “prime of life” group that reported an overall decrease in 0.01 of the mean value of SRHS in 2013 compared to 2000. No change was observed in the 45 to 64 years “middle age” group over time.

Additionally, we measured the income inequality ratio (20:20, that is the relative difference in mean health between extreme income quintiles) and the inequality gap (20–20, that is the absolute difference in mean health between extreme income quintile) in years 2000 and 2013 separately, and compared the change in 2013–2000 (Table 3). Both ratio and gap measures consistently found a decline in inequality over time (2013–2000) for all age categories except the very youngest age 15 to 24. Overall, for all ages and both sexes, adults in the richest fifth of households were 33% more likely than those in the poorest fifth to report above-average health in 2000, falling to 11% in 2013—a reduction in the inequality ratio of 22 percentage points down to a third of its previous level. In absolute gap terms, the proportion reporting above-average health was 19 percentage points higher in the richest fifth in 2000, falling to 7 percentage points higher in 2013 –a reduction in the inequality gap of 12 percentage points down to below one half of its previous level. Looking at the age-specific changes, the highest decrease in inequality was observed in the 75+ “elderly” age category (-0.78 in 20:20 change 2013–2000; -0.18 in the 20–20 change in 2013–2000). This means that the difference between top and bottom income groups in the proportion of elderly adults reporting “above-average” health decreased by 78% or 18 percentage points. For the 15 to 24 year “young adults” group, there was a corresponding increase in inequality in ratio and gap measures of 3% or 4 percentage points. All other age groups showed a decline in both ratio and gap measures of inequality.

**Table 3:** Inequality ratio (20:20) and inequality gap (20–20) of mean SRHS (range 0 “below average” to 1 “above average”) in years 2000 and 2013, by age groups.

	Year 2000				Year 2013				Change 2013–2000	
Age groups (years)	Poorest quintile	Richest quintile	*20*:*20 inequality ratio*	*20–20 inequality gap*	Poorest quintile	Richest quintile	*20*:*20 inequality ratio*	*20–20 inequality gap*	*20*:*20 2013–2000*	*20–20 2013–2000*
15 to 24 “young adults”	0.76	0.84	*1*.*10*	*0*.*08*	0.75	0.86	*1*.*14*	*0*.*11*	***0*.*04***	***0*.*03***
25 to 44 “prime of life”	0.62	0.86	*1*.*38*	*0*.*24*	0.59	0.76	*1*.*28*	*0*.*17*	***-0*.*10***	***-0*.*07***
45 to 64 “middle age”	0.34	0.67	*1*.*97*	*0*.*33*	0.35	0.56	*1*.*60*	*0*.*21*	***-0*.*37***	***-0*.*12***
65 to 74 “late middle age”	0.29	0.49	*1*.*68*	*0*.*20*	0.28	0.47	*1*.*67*	*0*.*19*	***-0*.*01***	***-0*.*01***
75+ “elderly”	0.24	0.53	*2*.*20*	*0*.*29*	0.26	0.37	*1*.*42*	*0*.*11*	***-0*.*78***	***-0*.*18***
**All ages**	**0.56**	**0.75**	***1*.*33***	***0*.*19***	**0.62**	**0.69**	***1*.*11***	***0*.*07***	***-0*.*22***	***-0*.*12***

#### The concentration curve and concentration index (CI)

**Fig 3:**
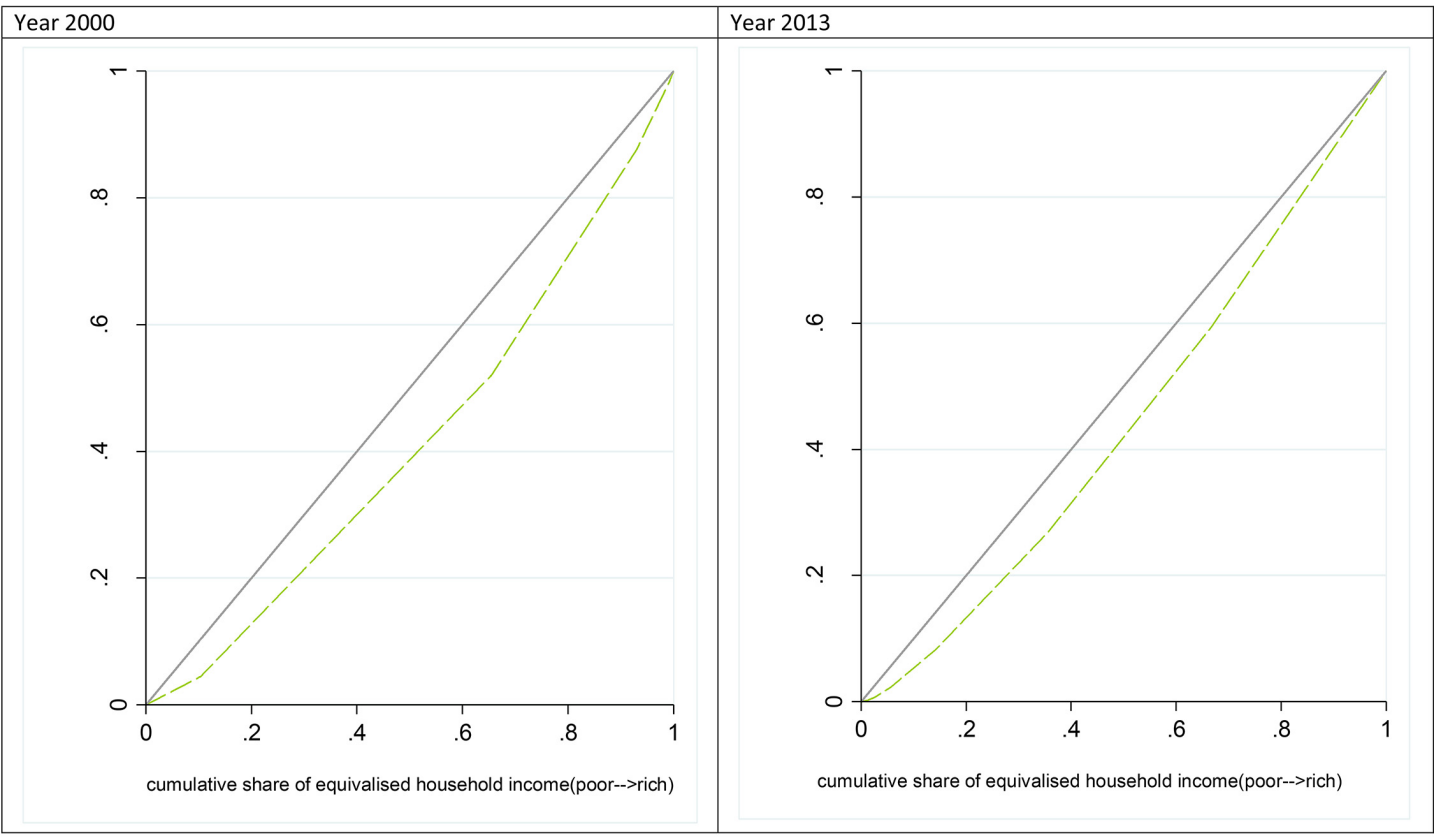
Concentration curves for SRHS in Chile before (2000) and after (2013) the healthcare reform in Chile of 2005.

Fig 3 displays the concentration curves for income related SRHS in years 2000 and 2013. Table 4 presents the results of the CI estimation for years 2000 and 2013 using different analytical methods. In year 2000, the uncorrected CI for SRHS in Chile by convenient regression method was 0.062 [SE = 0.0028]. The same measure in 2013 was 0.018 (SE = 0.0016). The corrected Erreygers CI estimation showed a positive value of 0.165 [SE = 0.0075] in 2000 and 0.047 [SE = 0.0088] in 2013. Despite some variation, all CIs values indicated that better health status was concentrated among the rich in Chile both before and after the equity-centered reform of 2005. Similar to the findings from the previous comparisons, this also suggests that inequality has decreased over time. Nevertheless, it should be noted that CI values are not on a ratio scale and therefore interpretation of the magnitude of change must be cautious.

**Table 4:** Concentration Indexes (CIs) for income related inequality in SRHS in Chile before (2000) and after (2013) the equity-centered healthcare reform of 2005.

	YEAR 2000	YEAR 2013
Uncorrected CI (SE)	0.062 (0.0028)	0.018 (0.0016)
Erreygers corrected CI (SE):	0.165 (0.0075)	0.047 (0.0088)
Uncorrected Horizontal Inequity Index (SE):	-	-
Directly standardized	0.051 (0.0008)	0.043 (0.0002)
Indirectly standardized w/o additive separability	0.071 (0.0026)	0.052 (0.0015)
Indirectly standardized with additive separability	0.051 (0.0008)	0.043 (0.0002)

#### The horizontal inequity index

Based on the standardization of the uncorrected CI, allowing its limitations for estimating the index for a bounded variable, the deviation of actual distribution from a fair one driven by legitimate determinants alone and in which all illegitimate differences have been removed was 0.051 [SE = 0.0008] in 2000 and 0.043 [SE = 0.0002] in 2013. The same results were obtained when we relaxed the additive separability assumption (Table 4). In every case, we observed a positive value of the CI. Both before and after the healthcare reform of 2005, we found that after removing legitimate factors associated with income inequality in SRHS in Chile, a concentration of above average SRHS favoring the rich over the poor persisted in Chile. Similarities observed between the uncorrected and the indirectly standardized CI estimates suggest a possible relatively small contribution of legitimate factors (i.e. a large contribution of illegitimate ones) on the degree of income-related inequality in SRHS in Chile in both 2000 and 2013. This was further explored through the decomposition approach of the corrected Erreygers CI, which is reported below.

#### Decomposition of the concentration index

**Fig 4:**
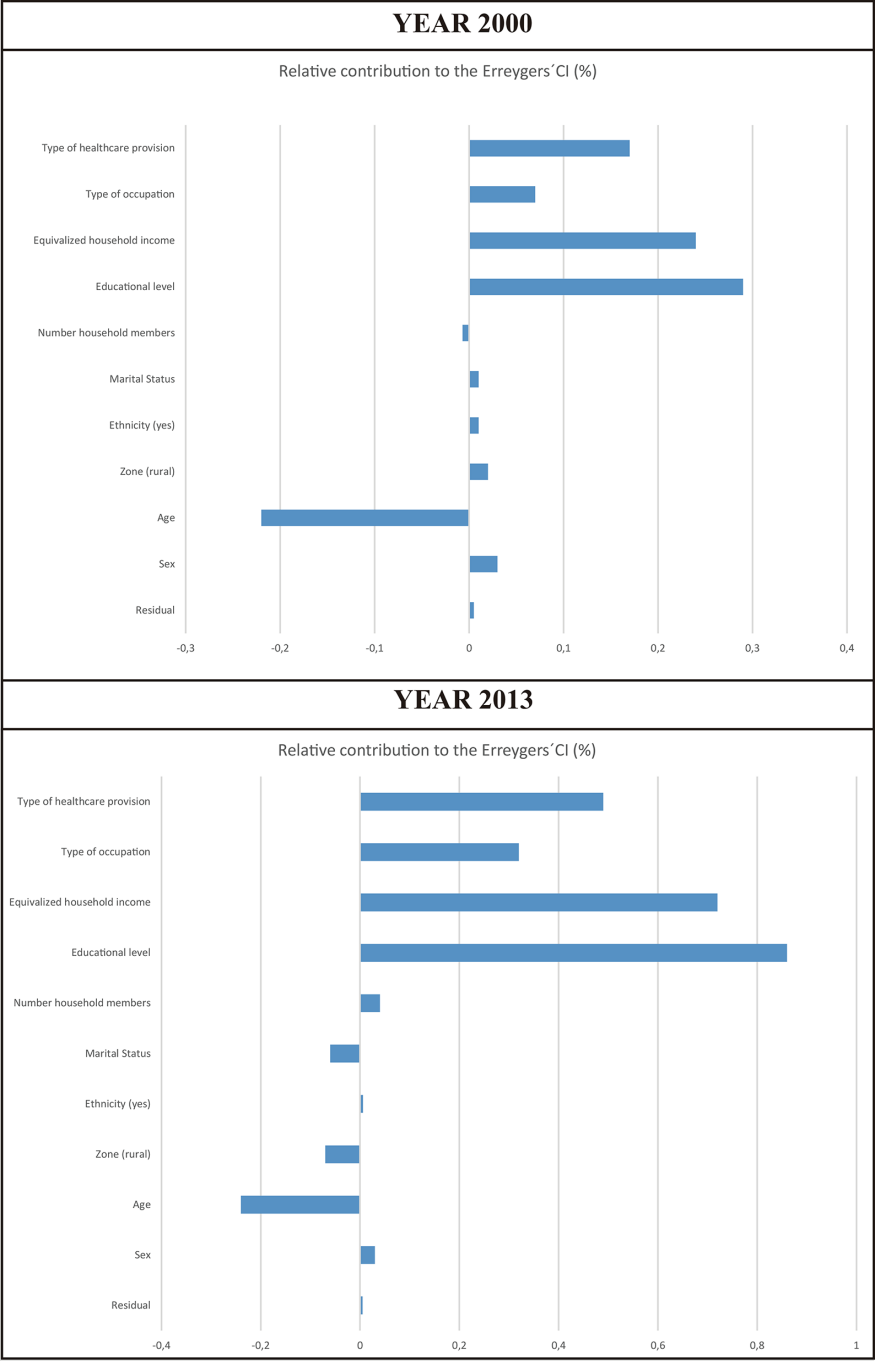
Decomposition of the Erreygers CIs: the relative contribution of each legitimate and illegitimate factor to inequality in SRHS in Chile before (2000) and after (2013) the healthcare reform in Chile of 2005*.

The largest contribution to inequality for both years came from illegitimate factors. In year 2000, most of the illegitimate inequality came from educational level, followed by equivalized household income and healthcare provision entitlement. Similarly, in year 2013 most of the illegitimate inequality came from educational level, income, and healthcare provision entitlement. Socioeconomic status, education and income in particular, persisted in being significantly associated with inequality in SRHS in Chile after the healthcare reform of 2005 and even increased its contribution to inequality in 2013 compared to year 2000. With the exception of age, legitimate factors were less relevant than illegitimate factors in general, but their significance decreased between 2000 and 2013. The absolute contribution from residuals (unexplained variation) was very low in both years (0.8% in 2000 and 3% in 2013) (Fig 4).

## Discussion

### Summary of key findings

There was a significant concentration of “above average” SRHS favoring rich people in Chile in both 2000 and 2013. Illegitimate factors were the largest contributors to income-related inequality in SRHS, largely explained by educational level, income, and healthcare provision entitlement. Age was the most significant legitimate factor, but its relevance decreased over time. Even though the overall degree of inequality in SRHS decreased after the equity-centered healthcare reform of 2005, inequality in SRHS associated with illegitimate factors persisted and grew between 2000 and 2013 in Chile. Additionally, the paper provides a detailed description of the demographic and socioeconomic picture of the adult population in years 2000 and 2013.

Illegitimate factors that contributed to income-related inequality in SRHS in Chile in 2000 and 2013 need special consideration, particularly education, income, and healthcare provision. Educational level has proven to be strong driver of human and economic development [50], as well as population wellbeing and life expectancy [51–53]. Chile is today facing a major educational reform that has the potential of improving both opportunity to higher education and quality of teaching. This study highlights the key role of education in population SRHS in Chile, as it reflects clear social gradients with this health outcome and a strong association with other relevant factors like income, type of occupation and healthcare provision entitlement [54].

Income is closely and strongly associated with health in Chile [25,55] and elsewhere [56]. Previous research highlights the multidimensional effects of poor income in health population [57,58]. Absolute poverty directly affects health, including self-reported health [59], through poor quality of neighborhood and household materials, insufficient heating during cold seasons and scarce money to buy good quality and healthy food, among others [60]. Relative poverty indirectly affects health, including subjective health [61], through psychosocial and cultural mechanisms related to social hierarchy, chronic stress and low self-esteem [62–64].

Healthcare provision in Chile is particularly interesting. As mentioned earlier in this manuscript, the Chilean healthcare system is a mixed system characterized by segmentation. The 2005 healthcare reform aimed at reducing social inequalities in access to diagnosis and treatment across the public and private sector, irrespective of age, sex, health status, or socioeconomic position. Based on this study´s findings, however, the effect of the healthcare system in Chile continues to be significantly associated to self-reported health. This occurs despite the subtle reduction in income-related inequality in self-reported general health in this adult population. The country might need more drastic changes in the healthcare system in coming years to truly affect population health in a more profound and effective way.

### Study strengths and weaknesses

This study uses a recent large national survey in Chile and robust measures of inequality based on both concentration index and quintile group approaches. It corrects for the bounded nature of the SRHS measure and provides weighted results in order to attain results that are as nationally representative as possible. There are, however, some limitations. First, about 15–20% of the population invited to participate in the surveys declined to do so, and there is no further information on their socio-demographic conditions or reasons for declining to participate. Second, institutionalized populations (e.g. prisons and hospitals) and a few geographically “hard to reach” boroughs in the country were excluded from this survey. If anything, this selection bias may make our results conservative, under-estimating the degree of income-related inequality, since institutionalized and “hard to reach” populations are likely to have both relatively low SES and below average SRHS. If so, this study provides a lower bound on the degree of income-related inequality in SRHS in Chile.

Third, the reliability of SRHS as a measure for assessing health can be misleading, since it depends on subjective health expectations and perceptions [65]. In particular, people with higher socioeconomic status may have higher health expectations and hence report lower SRHS for any given level of clinically assessed morbidity or health risk [66]. For this reason, use of SRHS could under-estimate the true degree of socioeconomic health inequality in a population.

This analysis was conducted using observational data, which is not designed for estimating causal effect. Besides, before and after designs have important limitations, in particular the lack of a control group. Given the absence of a control group we cannot assume that the observed difference between both periods can be explained, totally or partially, by the healthcare reform of 2005. Unfortunately, since the reform was implemented nationally, it is difficult to find a suitable control group.

A final limitation of our study was the change in the SRHS scale between year 2000 and 2013, which meant we were not able to measure change in absolute levels of health. However, we could still measure change in whether people reported themselves in “above average” or “below average” health. This means we were only able to measure change in relative inequality as opposed to absolute inequality. However, relative inequality is the most commonly used concept of inequality in the socioeconomic health inequality literature, and is the concept that underpins the concentration index approach. Furthermore, we have performed a sensitivity analysis to address this issue by comparing SRHS using a different cut-off point in year 2013 (S1 Table). This sensitivity analysis did not show relevant differences to the current analysis in the distributional effect of SRHS by ranked equivalized household income in the Chilean population. Since our focus is on relative health and not absolute health, we chose to define the cut-off point in terms of the average level of self-rated health in each year. By definition, the proportion of people reporting “above average” health cannot change much between the two years–indeed, overall this only changes by one percentage point (Tables 1 and 3). The analysis then focused clearly and exclusively on relative health comparisons by socioeconomic status–i.e. whether people are “above average” or “below average” in terms of self-rated health. This approach should minimize any potential bias associated with the change of scale and the associated artefactual increase in absolute levels of self-rated health in all socioeconomic groups.

### Interpreting our findings

Findings from this study are consistent with those of Vásquez et al. [25]. They measured income-related inequalities in health and health care utilization in the period 2000–2009 in Chile, including SRHS, and found that people in lower-income quintile groups report worse health status than people in higher income groups. Our study supports Vásquez [25] results and updates it with a longer period of time for comparison between measures and uses a more robust measure of income inequality.

What could explain the fall in the CI between 2000 and 2013? We must be cautious about interpreting our findings, for two main reasons. First, because the CIs are not ratio scale indices we cannot draw firm conclusions about how “large” or “small” the fall in inequality was. Second, this is a comparison between two cross-sectional surveys and we do not have any control groups or repeated measures on the same individuals or other robust ways of disentangling causal pathways. However, we explore some possible explanations below.

First, sustained economic growth, low unemployment, improved transport links and other general aspects of development outside the healthcare sector are relevant achievements for Chile in the past 20 years. This might explain some of the general improvement in SRHS. This might also explain more rapid improvement in health among poorer groups, to the extent that richer groups already had access to adequate nutrition, housing and other material conditions conducive to good health. Economic development could also reduce income inequality, which could further contribute to reduced income-related health inequality. As we can see from Table 2, the gap in household income between the wealthiest and the poorest quintiles decreased between years 2000 and 2013 (20:20 ratio of about 16 in 2000 and 11 in 2013). This must be taken into consideration when exploring the impact of the equity-based reform of 2005 in the SRHS of the total population, especially when not having a control group to compare to.

A second possible explanation could be changes in the age composition of the samples (mean age 30.6 years [95%CI = 30.4–30.8] in 2000; 35.3 years of age [95%CI = 35.1–35.5] in 2013, p<0.001) (Table 2). However, decomposition analysis showed that age was not the main contributor to the total variance of income inequality and even decreased over time.

Other possible explanations that we cannot fully test in this study are: (i) the existence of a systematic change in how the rich interpret and respond to this survey question (e.g. bias due to asymmetrically rising health expectations, rising faster among the rich than the poor), and (ii) change in the way the question on SRHS was framed. Differences between 2000 and 2013 in response categories on SRHS (5 categories versus 7 categories) might have differentially affected the distribution of responses in our sample by different population subgroups. The observed increase in the overall proportion of people reporting “above-average” SRHS between 2000 and 2013 was just 1 percentage point, from 57% to 58% (see Tables 1 and 3). It is possible that people are more likely to report health at or above the mean response when the scale has 7 points rather than 5 points. It is also possible that individuals with lower income may be more strongly influenced by any such bias than individuals with higher income. However, to invalidate our finding of a fall in inequality, any such bias would have to be (a) positive, (b) substantial, and (c) systematically larger for poorer individuals than richer individuals. We are not aware of any evidence that all three conditions hold.

It must be noted that our study does not aim to measure change in absolute levels of health; it aims to measure change in relative levels of health between people with different socioeconomic status–i.e. socioeconomic health inequality. The change in the health measurement scale between the two time points means that we cannot study change in population average health but we can study change in socioeconomic health inequality. Reducing socioeconomic health inequality is an important health policy objective and our study provides relevant information about this significant issue that can inform other similar emerging and developed economies. Future research on this subject could advance this work by attempting to identify the causes of change and ideally using longitudinal data if available.

## Conclusions

The analyses presented in this manuscript answers both research questions: (i) to what extent was above average SRHS in the adult Chilean population concentrated upon individuals with higher ranked equivalized household income in the years 2000 and 2013?; and (ii) what were the absolute and relative contributions of legitimate and illegitimate determinants of income-related inequality in SRHS in 2000 and 2013?

In order to answer research question one we measured income-related health inequality in 2000 and 2013; and to answer research question two we provided a decomposition analysis showing the contribution of legitimate and illegitimate factors to income-related health inequality in both periods. We found that “below average” SRHS in the Chilean population in 2000 and 2013 was significantly concentrated on adults from relatively low-income households. Income-related inequality in SRHS appeared to have fallen between 2000 and 2013, although the contribution of illegitimate factors to income-related inequality in SRHS remained large over time.

Future research could attempt to validate our findings using other indicators of health, such as mortality. It could also analyze broader contextual determinants of income-related health inequality in Chile and elsewhere, such as working conditions, perceived control and autonomy at work, self-perceived SES, discrimination, and social capital components. This first study on health inequality in Chile before and after the 2005 reform suggests that policies to extend universal healthcare coverage can make an important contribution to reducing socioeconomic health inequality, but that broader cross sectoral policies are also needed. This study suggests that policymakers continue to face substantial challenges in tackling socioeconomic health inequality in Chile, despite nearly a decade of the equity-oriented healthcare reform of 2005, and suggests that broader cross sectoral policies may also be required.

## Supporting Information

S1 Table Concentration Indexes (CIs) for income related inequality in SRHS in Chile after (2013) the equity-centered healthcare reform of 2005 using a different cut-off point (deterministic sensitivity analysis). (DOC)
