# Supplementary material for: Did Socioeconomic Inequality in Self-Reported Health in Chile Fall after the Equity-Based Healthcare Reform of 2005? A Concentration Index Decomposition Analysis
Source: PLoS One. 2015 Sep 29;10(9):e0138227. doi: 10.1371/journal.pone.0138227 (PMC4587959; doi:10.1371/journal.pone.0138227)
Supplement: S1 Table — (DOC) [file pone.0138227.s001.doc]

**S1 Table** Concentration Indexes (CIs) for income related inequality in SRHS in Chile after (2013) the equity-centered healthcare reform of 2005 using a different cut-off point (deterministic sensitivity analysis)

|  | **YEAR 2013** | |
| --- | --- | --- |
|  | Measure used in this analysis:  Below average SRSH “0”: 1-5  Above average“1”: 6-7 only | Sensitivity analysis: Narrower measure of poor SRHS:  Below average SRHS “0”: 1-4 only  Above average SRHS “1”: 5-7 |
| Uncorrected CI (SE) | 0.018 (0.0016) | 0.014 (0.0009) |
| Erreygers corrected CI (SE) | 0.047 (0.0088) | 0.048 (0.0044) |
